# Glycan Activation of Clec4b Induces Reactive Oxygen Species Protecting against Neutrophilia and Arthritis

**DOI:** 10.3390/antiox11010012

**Published:** 2021-12-22

**Authors:** Mike Aoun, Xiaojie Cai, Bingze Xu, Gonzalo Fernandez Lahore, Michael Yi Bonner, Yibo He, Liselotte Bäckdahl, Rikard Holmdahl

**Affiliations:** 1 Department Medical Biochemistry and Biophysics, Division Medical Inflammation Research, Karolinska Institute, 171 77 Stockholm, Sweden; mike.aoun@ki.se (M.A.); xiaojie.cai@ki.se (X.C.); bingze.xu@ki.se (B.X.); gonzalo.ariel.fernandez.lahore@ki.se (G.F.L.); michael.bonner@ki.se (M.Y.B.); yibo.he@ki.se (Y.H.); liselotte.backdahl@ki.se (L.B.); 2 Department of Cardiovascular Medicine, The First Affiliated Hospital of Xi’an Jiaotong University, Xi’an 710061, China; 3 The Second Affiliated Hospital of Xi’an Jiaotong University, Xibei Hospital, Xi’an 710004, China

**Keywords:** glycan, C-type lectin receptor, NADPH oxidase 2, neutrophilia, arthritis

## Abstract

Animal models for complex diseases are needed to position and analyze the function of interacting genes. Previous positional cloning identified Ncf1 and Clec4b to be major regulators of arthritis models in rats. Here, we investigate epistasis between Ncf1 and Clec4b, two major regulators of arthritis in rats. We find that Clec4b and Ncf1 exert an additive effect on arthritis given by their joint ability to regulate neutrophils. Both genes are highly expressed in neutrophils, together regulating neutrophil availability and their capacity to generate reactive oxygen species. Using a glycan array, we identify key ligands of Clec4b and demonstrate that Clec4b-specific stimulation triggers neutrophils into oxidative burst. Our observations highlight Clec4b as an important regulator of neutrophils and demonstrate how epistatic interactions affect the susceptibility to, and severity of, autoimmune arthritis.

## 1. Introduction

Rheumatoid arthritis (RA) is an autoimmune disease that affects 0.5 to 1% of the population and if untreated often leads to irreversible joint damage [1]. RA is a complex disease due to the intricate interplay between genetic and environmental factors. Genome-wide association studies identified several major histocompatibility complex (MHC) and non-MHC loci associated with RA. However, these variants explain only a limited degree of the estimated heritability [2] and very few, if any, of the underlying genes have been identified and their functional role elucidated. Animal models of complex diseases, such as arthritis, offer a possibility not only to position the underlying polymorphism but also to study their pathogenic role in a controlled setting [3]. A single intradermal injection at the tail base of pure adjuvants such as pristane is enough to induce a severe arthritis-like phenotype (pristane-induced arthritis, PIA) in many rat strains [2]. Linkage studies performed by our lab and others in F2 crosses between a resistant inbred strain (i.e., E3 or PVG) and a susceptible counterpart (i.e., DA) identified the major quantitative trait loci (QTL) that regulate clinical score, onset and paw swelling in PIA [4,5,6,7,8,9]. PIA4 and PIA7 QTLs, on chromosome 12 and chromosome 4 respectively, are the strongest regulators of multiple arthritis models in rats [10]. Positional cloning of PIA7 and PIA4 have identified Clec4b and Ncf1 as the regulating genes for arthritis severity and onset as well as controlling innate and adaptive immune responses.

Ncf1 codes for neutrophil cytosolic factor 1 protein (also denoted p47phox), a member of the nicotinamide adenine dinucleotide phosphate (NADPH) oxidase complex. Rats with the DA Ncf1 alleles have a single nucleotide polymorphism (SNP) in codon 153 with an amino acid variant (T153M) without affecting protein expression [6]. The DA SNP allele results in a significant decrease in NADPH oxidase activity whilst increasing arthritis susceptibility and severity [6]. The exact mechanism of how this reduction leads to an amplified disease score is likely to be complex and may vary in different diseases. The Ncf1/NOX2 complex will regulate not only antigen presentation [11] and T cell function [12] but also the inflammatory status of phagocytes [13]. Remarkably, two independent reports showed a missense variant in Ncf1 in humans associated with systemic lupus erythematosus (SLE), Sjögren’s syndrome, and RA. Moreover, a decreased copy number of Ncf1 predispose to SLE and RA, while the reverse protects against the disease [14,15].

Clec4b codes for the dendritic cell immune-activating receptor, earlier denoted as Dcar. A missense SNP in DA rats on chromosome 4 introduces a stop codon in the second exon of Clec4b gene, which is inherited together with a likely conserved haplotype containing a family of closely related C type lectin receptors (CLR). Clec4b is a member of the group II CLR superfamily, which are cell-specific, highly conserved among species and bind conserved moieties of exogenous or endogenous origin [16]. Even though CLRs are mainly studied in infection models [17,18,19], they are engaged also in allergy [20] and autoimmune models [21,22]. We recently showed that the abrogation of Clec4b protein in dendritic cells increased PIA severity and incidence, and it skewed T cell proliferation, activation, and Th17 subset expansion [23].

In response to various ligands, most CLRs induce the production of cytokines and reactive oxygen species (ROS) [24] as a defense mechanism against pathogen. We now show that Clec4b is strongly expressed on neutrophils and found to be associated with neutrophilia and cytokine production in models of inflammation. We present two novel insights of the role of Clec4b in autoimmune disease: (1) interaction between Clec4b-Ncf1 manifests as an additive effect on PIA clinical outcome and (2) stimulation via Clec4b induces ROS production through the Ncf1/NOX2 complex, an activation pathway shown to be of major regulatory impact on the development of the inflammatory response and arthritis.

## 2. Materials and Methods

### 2.1. Animals

DA/ZtmRhd (DA) and E3/ZtmRhd (E3) were originally obtained from Zentralinstitut fur Versuchstierkunde (Hannover, Germany) but maintained as inbred strains in our laboratory for more than 20 generations. Clec4b and Ncf1 congenic strains were produced by marker-assisted selective breeding and contain a DA background genome except in the congenic interval containing E3 allele. The Clec4b congenic harbors a 200 Kb fragment between 164.71 Mb and 164.91 Mb according to the Baylor Rn4 2004 assembly and the Ncf1 congenic has been described previously [6]. Animals were maintained at the Scheele Laboratory, Karolinska Institutet in a pathogen-free environment according to the Federation of European Animal Laboratory Science Association guidelines (FELASA). All procedures and animal experiments have been approved by the local ethics committee (Stockholms djurförsöksetiska nämd/Ethical approval number N35/16 for CIA/PIA and OIA while N181/13 for air pouch experiments). The animals were maintained in a 12 h light and 10 h dark cycles and held individually in cages containing wood shavings and aspen. Moreover, they had free access to water and were fed with the standard rodent chow.

### 2.2. Arthritis Experiments

Arthritis was initiated by a single intradermal injection at the dorsal side of the tail base. For PIA induction, 150 μL pristane (2,6,10,14-tetramethylpentadecane, 95%; Acros Organics, Morris Plains, NJ) was injected. For induction of OIA, 150 μL mineral oil (incomplete Freund’s adjuvant /IFA/) (BD, USA) was injected. For induction of CIA, rat collagen type II (CII), dissolved in 0.1M acetic acid and emulsified in IFA was injected. All used rats were older than 8 weeks old. All experiments were performed with littermate rats that are age-matched, distributed within the cages and blindly evaluated by the investigator. The rats were daily inspected which involves monitoring the limbs for arthritis development by a previously described macroscopic scoring system [25]. Briefly, 1 point was given for each individual swollen and erythematic toe and up to 5 points for an inflamed ankle (15 points in total per paw). The scoring was carried out every other day for 20–30 days after disease induction. No points were given to deformed paws that did not exhibit signs of edema.

### 2.3. Carrageenan Air-Pouch Model

Air pouches were formed in 10- to 12- weeks old rats according to the described protocol [26]. Briefly, 5 to 6 mL of sterile air was injected in the region between the scapulae of the animals. 3 days later, 2 to 3 mL of air was reinjected in the same spot to reconstitute the pouch structure. Induction of the inflammation was carried out by injecting at day 6, 1 mL of 1% λ-Carrageenan (Sigma-Aldrich) solution and rats were sacrificed 1 to 3 h later. Inflammatory exudate as well as air pouch cells were harvested using a 5.5 mM EDTA (Sigma-Aldrich) solution. Cells were processed by flow cytometry and cytokine concentrations were determined by sandwich ELISA.

### 2.4. Gene Expression

RNA was extracted from −80 °C snap frozen tissue samples using RNAeasy (Qiagen, Hilden, Germany) cDNA was prepared by reverse transcriptase (iScript Bio-Rad Hercules, California, CA, USA) and qPCR amplification was performed using iq-SYBR green supermix (Bio-Rad Hercules, California, CA, USA). To eliminate potential splicing effects, two different primer-sets that cover different regions of the Clec4b gene (NCBI Gene ID: 450222), were employed. Clec4b.1F-AAAACTGCCCCAAGGTAAGG, Clec4b.1R—GAAGCAGGTGCTG AGGAGTAA, Clec4b.2F-TGCTCATCTGTTGGTGATCCA, Clec4b.2R-TGTAAAATAACCCCAACGAGTGTCTA.

### 2.5. Histology

Rats were euthanized at the end of CIA and PIA. Skinless hind paws were fixed for a week in 4% phosphate-buffered formaldehyde, followed by decalcification using EDTA and later embedded in paraffin. Sections of tissues were stained with hematoxylin/eosin and scored blindly. Histological scores were assessed for synovium, joint, cartilage and bone architecture. For synovium, 0–2 points were given to each of the following criteria: severity, hyperplasia, infiltration, angiogenesis, pannus formation, and necrosis. For cartilage erosions, 0–2 points were given to each of the following criteria: erosion and new formation. For bone erosions, 0–2 points were given to each of the following criteria: new bone formation and erosion. For joint destruction, 0–6 points were given to each of the following criteria: joint destruction and ankylosis. The score for synovium (10), cartilage (4), bone (4), and joint structure (12) add up to 30 points. In addition, the tissue sections were stained with Safranin O to study cartilage destruction.

### 2.6. Glycan Array

Glycan array 100 was purchased from RayBiotech (USA) and the assay was performed according to the manufacturer’s protocol. Briefly, Glycan sub arrays were blocked for 30 min using diluent provided by the kit. Next, Clec4b proteins were incubated with glycans immobilized on a glass slide for 3 h. Sub-arrays were washed followed by incubation with biotinylated anti-histidine mAb (Thermo Fisher Scientific) for 2 h. Sub-arrays were washed and incubated with Cy-3 labelled streptavidin for 1 h. The glass slides were scanned with a gene microarray laser scanner and fluorescence values were obtained and analyzed.

### 2.7. Flow Cytometry and Fluorescence-Activated Cell Sorter

Single cells suspensions were carried out in ice-cold fluorescence-activated cell sorting buffer (Ca2+-and Mg2+-free Dulbecco’s PBS supplemented with 1% fetal calf serum, 2 mmol/L EDTA) and blocking was presumed using 2.4G2/BD Fc Block (CD32) antibody. Subsequently, cells were stained with a saturating concentration of monoclonal antibodies on 96-well V-bottom polypropylene plates (BD Biosciences). The following antibodies were used: CD45 (OX-1), CD11b (WT.5) and MCP-1 was purchased from BD Biosciences (San Diego); CD103 (OX-62), CD11b/c (OX-42), CD4 (OX-35) was purchased from BioLegend; αβTCR (R-73), CD45R/B220 (His-24), MHC-II (OX-17) were purchased from eBioscience (San Diego); CD68 (ED-1) was purchased from AbD Serotec and Dcar-Fitc (Wen-41) mAb was a kind gift from Sigbjørn Fossum/Erik Dissen. After extracellular staining, cells were incubated in fixation/permeabilization buffer (eBioscience) and washed with permeabilization buffer (eBioscience) before intracellular staining of CD68 and MCP-1. Both LIVE/DEAD Violet (Invitrogen, Carlsbad, CA, USA) and the forward scatter vs. side scatter plot were used to include only live/singlets. A SORP BD LSR II analytic flow cytometer (BD Biosciences) was used for acquisition, and the data were analyzed with FlowJo software version 8.8.6 (Tree Star, Ashland, OR)

For splenic neutrophil sorting, single cells suspensions from rat spleens were carried out as described above and cells were stained with monoclonal antibodies. Neutrophils (CD11b/c^+^ His48^+^) were sorted using the MoFlo XDP cell sorter (Beckman Coulter) while T cells were magnetically isolated using the Pan T cell Microbeads (Miltenyi Biotec) according to manufacturer’s protocol. RNA was extracted from both samples and gene expression analysis using SYBR green followed.

The protocol for the ligand detection using bead-based assay was based on a previously described protocol with minor changes [27]. Briefly, compensation beads (BD Biosciences) were coated with anti-histidine mAb (Thermo Fisher Scientific). Subsequently, Dcar-EPN, Dcar-QPD and a decoy protein was used to couple it to the beads via the polyhistidine-tag and the whole complex was incubated with FITC-tagged Zymosan bioparticles (Thermo Fisher Scientific) at different concentration. Beads were gated, and non-singlets were excluded using FSC-A vs. SSC-A followed by determination of geometric mean fluorescence intensity of FITC channel.

### 2.8. Extracellular and Intracellular ROS Detection

For flow cytometry-based detection of intracellular ROS, blood and BM cells were incubated with 3 µM dihydrorhodamine (DHR) 123 (Invitrogen) and stimulated (20 min, 37C) with 200 nM PMA (Sigma-Aldrich), 3µM Fmlp (Sigma-Aldrich), 250 µg/mL Zymosan (Sigma-Aldrich) and absence of stimuli. Geometric mean fluorescence intensity of rhodamine 123 was corrected for background fluorescence (dimethyl sulfoxide) and fold increase was determined. For chemiluminescence-based detection of extracellular ROS, BM cells were plated in duplicates and stimulated with 200 nM PMA, 3 µM Fmlp, 500 µg/mL Zymosan and absence of stimuli. Next, extracellular ROS was measured chemiluminescence assay in HBSS++ containing 150 μM isoluminol/18.75 U/mL HRPII. Data output was measured in relative light units (RLU), and total RLU integrated over time (90 min) was plotted.

### 2.9. Expression and Purification of the Rat Dcar and Its Mutant

For expression of the proteins in mammalian cells, the molecules were designed as shown in Table 1 below. The signal sequence was from the human myeloid cell surface antigen CD33, followed by a polyhistidine-tag, a thrombin cleavage site and the extracellular domain of the protein. Eurofins synthesized the genes with KpnI and XhoI restriction sites at the 5′ and 3′ ends. The synthesized genes were restriction enzymes digested using FastDigestTM enzymes (ThermoFisher Scientific). The DNA fragments obtained were ligated into a mammalian expression vector pCEP4 (ThermoFisher Scientific) digested using the same restriction enzymes. After sequence verification, the plasmids were transfected into Expi293FTM cells (ThermoFisher Scientific) using the FectoPROTM DNA transfection reagent (Polyplus transfection). The supernatants were harvested 6 days post transfection. The recombinant protein was captured using a 5 mL HisTrap Excel (Cytiva) affinity column followed by size exclusion chromatography on a HiLoad Superdex 200 pg column (Cytiva). The purified protein was obtained as a single peak, concentrated and diafiltrated into PBS using an Amicon centrifuge device with a MWCO of 10 kDa. The protein concentration was determined by absorbance at 280 nm and the purity was analyzed by SDS-PAGE under both reducing and non-reducing conditions (Figure S1). For polyhistidine-tag control, a non-related protein from another project was used.

### 2.10. Cytokine Quantification

IL-6, TNF-α and IL-1β concentrations from the air pouch exudates were quantified using sandwich-based ELISA according to the manufacturer’s protocol (DuoSet kits, RnD systems). Serum from CIA rats was quantified for cytokines using a Bio-plex rat cytokine and chemokine assay (Luminex technology, Bio-Rad).

### 2.11. Statistical Analysis

Data were analysed using Graphpad Prism software (version 8.0.2) and information on the specific statistical test performed is included in all figure legends.

## 3. Results

### 3.1. Clec4b Regulates Collagen Induced Arthritis and Has a Profound Effect on Cytokine Production

**Figure 1:**
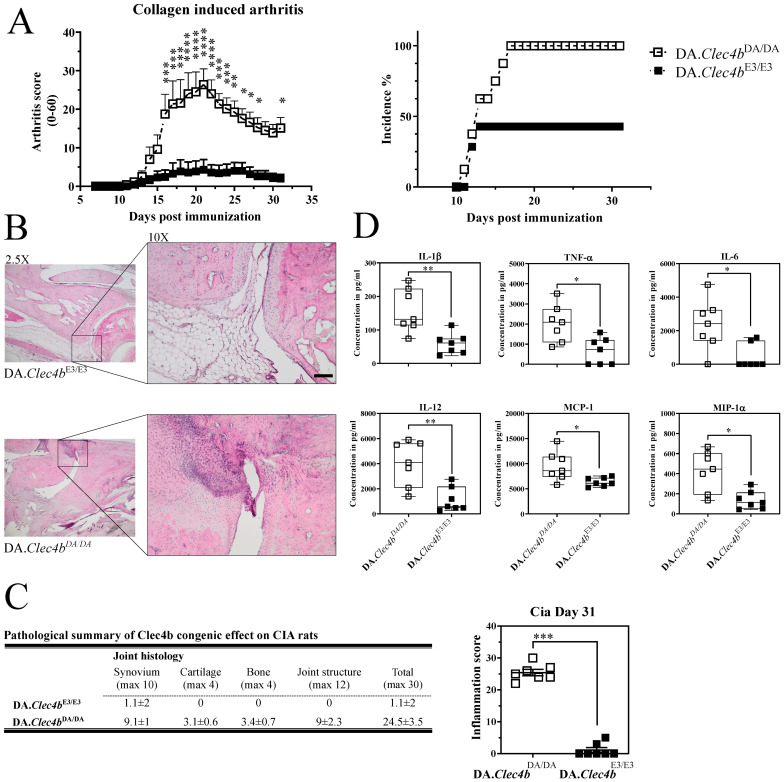
Clec4b congenic rats are protected against collagen type II-induced arthritis. Development of CIA in DA.Clec4bDA/DA (*n* = 7) rats compared with DA.Clec4bE3/E3 (*n* = 7) congenic rats. Rats were immunized with 100 µg of rat collagen type II emulsified in incomplete Freund’s adjuvant intradermally at the tail base. All rats were sacrificed 31 days post immunization for histology and blood sampling. (**A**) CIA clinical score and incidence plotted against days post immunization. (**B**) Representative histological samples of ankle joints taken at CIA end-point. Sections were stained with hematoxylin and erythrosine (2.5× and 10×). Scale bar represents 100 mm. Severe synovial and bone destruction is seen in DA.Clec4bDA/DA rats, whereas DA.Clec4bE3/E3 are presented with very mild bone and synovial destruction. (**C**) Serum samples collected from both congenic rats at CIA end-point and further quantified for cytokines and chemokines with luminex. (**D**) Histological slides were scored for synovial, cartilage, bone and joint structure and an inflammation score was plotted against different rat genotypes. Arthritis scores are presented as mean with SEM. Statistics in (**A**) determined using two-way analysis of variance with Sidak multiple comparison test; in (**B**) multiple *t*-tests non-parametric; in (**D**) two-tailed Mann–Whitney U test. A representative experiment of 2 independent experiments is shown. * *p* < 0.05; ** *p* < 0.01; *** *p* < 0.001; **** *p* < 0.0001.

To confirm the influence of Clec4b on arthritis and further characterize its effect on the inflammatory response, we chose the classical collagen-induced arthritis (CIA) model. We used congenic DA rats, expressing different alleles of Clec4b (susceptible DA and resistance E3), thereby denoted as DA.Clec4bE3/E3 and DA.Clec4bDA/DA respectively. As expected, the DA.Clec4bE3/E3 rats showed a milder disease with lower incidence as compared with the wild type (WT) DA.Clec4bDA/DA rats (Figure 1A). Histological staining of DA.Clec4bDA/DA joints showed enhanced destruction of cartilage, bone, and joint structure as well as leukocyte infiltration in the synovium when compared to DA.Clec4bE3/E3 joints (Figure 1B,C). Moreover, the concentration of pro-inflammatory innate cytokines and chemokines was found to be markedly higher in the serum of DA.Clec4bDA/DA rats (Figure 1D), implicating a role in regulating cytokines.

### 3.2. Clec4b Is Strongly Expressed on Neutrophils and Its Abrogation Leads to an Expansion of Neutrophils in Lymphoid Organs

**Figure 2:**
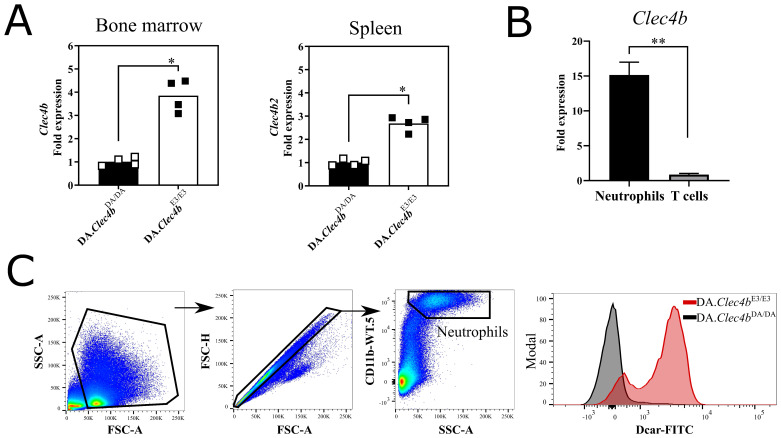
Clec4b is expressed mainly on myeloid cells. (**A**) Clec4b expression level as determined by quantitative real-time PCR (qRT-PCR) on naive bone bone marrow and spleen samples derived from DA.Clec4bDA/DA and DA.Clec4bE3/E3 rats (*n* = 4/group). (**B**) Clec4b expression level determined by qRT-PCR on FACS splenic neutrophils (*n* = 3) and T cells (*n* = 2) derived from DA.Clec4bE3/E3 rats. (**C**) Representative flow cytometry plots showing Clec4b expression on neutrophils derived from DA.Clec4bDA/DA and DA.Clec4bE3/E3 spleens (*n* = 4/group). Statistics in (A) were determined using two-tailed Mann-Whitney *U* test; in (**B**) determined using Welch’s *t* test. * *p* < 0.05 and ** *p* < 0.01.

**Figure 3:**
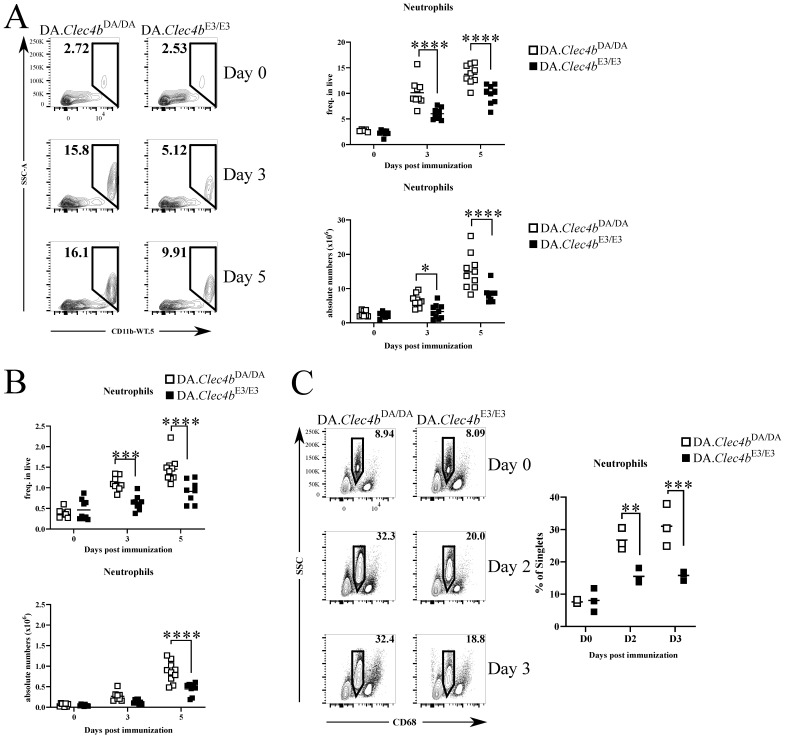
DA.Clec4bDA/DA rats display increased neutrophils accumulation in lymphoid organs during early phase of arthritis. (**A**) Flow cytometry plots followed by frequencies and absolute numbers of gated neutrophils (Singlets/Live/CD11b^+^ SSC^high^) in the spleens of DA.Clec4bDA/DA and DA.Clec4bE3/E3 rats at indicated time points after immunization. (**B**) Frequencies and absolute numbers of neutrophils in the lymph nodes collected at indicated time points after immunization. (**C**) Flow cytometry plots followed by frequencies of gated neutrophils (Singlets/Live/CD68^low^SSC^high^) in the blood of DA.Clec4bDA/DA and DA.Clec4bE3/E3 rats at indicated time points after immunization. Data in (**A**) and (**B**) are pooled from two experiments with *n* = 8–10/genotype. Data in (**C**) are representative of two independent experiments with *n* = 3 rats/group. Statistics in (**A**–**C**) determined using two-way analysis of variance with Sidak multiple comparison test. * *p* < 0.05; ** *p* < 0.01; *** *p* < 0.001; **** *p* < 0.0001.

Next, we analyzed the expression of Clec4b in the bone marrow (BM) and spleen by qPCR (Figure 2A). Fluorescently activated cell sorted splenic neutrophils (Figure 2B) were found to express readily Clec4b at a steady-state level. Using a rat Clec4b specific monoclonal antibody (clone: Wen41) [28], we confirmed by flow cytometry the protein expression on neutrophils (Figure 2C). We have earlier shown that the expression of Clec4b on CD4+ dendritic cells are regulating the activation of autoreactive T cells [23] but since the strongest expression is on neutrophils that play an important role in the development of arthritis [29,30], we now suggest an additional Clec4b-mediated neutrophil function. Therefore, we immunophenotyped the lymphoid organs (mainly spleen and lymph nodes) during the first 3–5 days immunization. The combination of monoclonal CD11b antibody (WT.5 clone) and side scatter allowed us to delineate and enumerate neutrophils in spleens and LNs of rats immunized with pristane. The frequency and absolute number of neutrophils in both lymphoid organs of immunized rats differed drastically between genotypes (Figure 3A,B). Even early blood samples from immunized DA.Clec4bDA/DA rats had almost double the frequency of neutrophils compared to DA.Clec4bE3/E3 rats (Figure 3C); this suggests a Clec4b-mediated mechanism in controlling neutrophilia during the early stage of arthritis. In short, we show that loss of Clec4b results in an uncontrollable expansion of neutrophils following an immune response and the increased accumulation of neutrophils in lymphoid organs, notably spleen and LN.

### 3.3. Clec4b-Ncf1 Interaction Shows an Additive Effect on Arthritis

**Figure 4:**
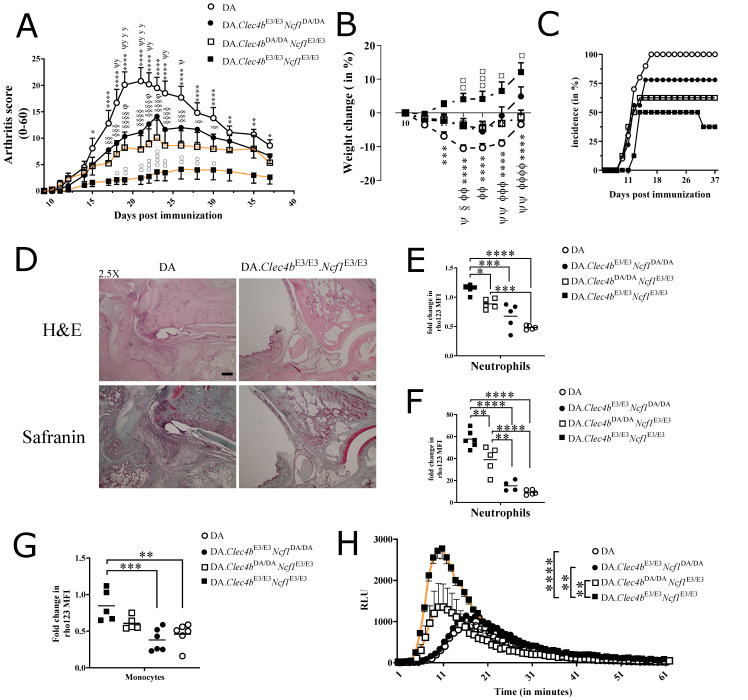
Additive effect of Clec4b and Ncf1 on arthritis clinical score. Development of PIA in DA.Clec4bDA/DA.Ncf1E3/E3, DA.Clec4bE3/E3.Ncf1DA/DA, DA.Clec4bE3/E3.Ncf1E3/E3 and DA controls (*n* = 8–10 rats/group). Rats were immunized with 150 µL of Pristane intradermally at the tail base. Rats were scored every other day (**A**) and weighted twice a week (**B**) throughout the disease course. (**C**) Incidence of the 4 groups of rats plotted against days post immunization. (**D**) Representative histological samples of ankle joints taken at PIA end-point. Sections were stained with hematoxylin/erythrosine (H&E) and safranin stain. Severe synovial and cartilage destruction is seen in DA controls, whereas DA.Clec4bE3/E3.Ncf1E3/E3 rats are presented with intact bones (talus and tibia). Scale bar represents 25 mm. Neutrophils (CD11b/c^+^His48^+^) from bone marrow (**E**) or blood (**F**) of 4 rat groups were analyzed for intracellular PMA-induced ROS production determined by DHR assay (*n* = 4–6 rats/group). (**G**) Monocytes (CD11b/c^+^His48^-^) from naive bone marrow of all rat genotypes were analyzed for intracellular PMA-induced ROS production determined by DHR assay (*n* = 5–6 rats/group). (**H**) chemiluminescence detection of extracellular ROS in PMA-primed bone marrow cells from all rat groups (*n* = 4–6 rats/group). Data in (A, B and H) are presented as mean with SEM and horizontal lines in (**E**–**G**) represent mean values. Symbols shown in (**A**,**B**): * DA.Clec4bE3/E3.Ncf1E3/E3 significant vs. DA; § DA.Clec4bDA/DA.Ncf1E3/E3 significant vs. DA; ψ DA.Clec4bE3/E3.Ncf1DA/DA significant vs. DA; φ DA.Clec4bDA/DA.Ncf1E3/E3 significant vs. DA.Clec4bE3/E3.Ncf1E3/E3; DA.Clec4bE3/E3.Ncf1DA/DA significant vs. DA.Clec4bE3/E3.Ncf1E3/E3. One symbol/star *p* < 0.05; two symbols/stars *p* < 0.01; three symbols/stars *p* < 0.001; four symbols/stars *p* < 0.0001. Statistics in (**A**,**B**) determined using two-way analysis of variance with Tukey’s multiple comparison test; in (**E**–**H**) determined using one-way analysis of variance with Tukey’s multiple comparison test. All data are representative experiments of at least 3 independent experiments.

To understand how the two major positionally cloned arthritis-regulating genes manifest on rat models of arthritis, we crossed DA.Clec4bE3/E3 with DA.Ncf1E3/E3 congenic rats. We introgressed single (Clec4b) or double (Clec4b/Ncf1) alleles derived from arthritis-resistant E3 rat strain into the arthritis-susceptible DA strain. Because both genes contribute to PIA, we used both single congenic rats (DA.Clec4bE3/E3.Ncf1DA/DA and DA.Clec4bDA/DA.Ncf1E3/E3), double congenic rats (DA.Clec4bE3/E3.Ncf1 E3/E3), and WT controls (DA). All 4 sub-congenic strains were followed daily throughout PIA disease course. Single congenic rats scored comparably in terms of disease severity (score of 10–15 at 21 dpi), incidence (62.5% and 78% for DA.Clec4bDA/DA.Ncf1E3/E3 and DA.Clec4bE3/E3.Ncf1DA/DA, respectively) and weight loss (4% on average at 21 dpi). In contrast, 100% of DA control rats developed arthritis (score of 20 at 21 dpi), while double congenic rats DA.Clec4bE3/E3.Ncf1E3/E3 remained resistant (less than 50% incidence) with very mild score (score of 5 at 21 dpi) and gained instead of losing weight (Figure 4A–C). This argues for an additive effect on disease severity, weight loss, and incidence. Representative histological slides of the arthritis prone DA rats revealed increased cartilage destruction (Safranin stain) and synovial tissue leukocytes infiltration (H&E stain) compared to arthritis resistant double congenic rats (Figure 4D).

### 3.4. Clec4b-Ncf1 Additive Interaction on Extracellular and Intracellular ROS Production

Upon stimulation, cytosolic Ncf1 is phosphorylated and translocated to the membrane where it assembles with other adaptor proteins (CYBA, CYBB, NCF2 and NCF4, Rac) of the NOX2 complex. Of note, complete deficiency in any of the above-mentioned genes can cause severe granuloma in several organs leading to chronic granulomatous disease (CGD) [31]; whereas partial deficiency of ROS increases the susceptibility to various autoimmune diseases [14,32]. To test whether stimulation of Clec4b generates NOX2-dependent ROS, we measured intra- and extracellular levels of ROS in blood and BM cells subjected to different stimuli. PMA stimulation of blood and BM cells resulted in the induction of ROS production both intra- and extracellularly, inversely mimicking the clinical phenotype. These observations advocate for a mechanistic role involving the Clec4b-ROS axis in regulating arthritis. Furthermore, intracellular ROS present in BM and blood neutrophils were 0.5 to 20 folds, respectively, lower in DA.Clec4bDA/DA.Ncf1E3/E3 compared to DA.Clec4b E3/E3.Ncf1E3/E3 congenic rats (Figure 4E,F). We did not observe any major changes between DA.Clec4bE3/E3.Ncf1DA/DA and DA controls, possibly due to the low functionality of the Ncf1DA allele. Additionally, monocytes appeared to be indifferent to the loss of Clec4b as no major changes in ROS production could be noticed (Figure 4G). Extracellular ROS was also investigated and as expected, ROS levels were almost 2 times higher in DA.Clec4bE3/E3.Ncf1E3/E3 compared to DA.Clec4bDA/DA.Ncf1E3/E3 (Figure 4H). In conclusion, stimulating Clec4bE3/E3 and Clec4bDA/DA cells with broad stimuli, such as PMA, manifested into a disparate ROS profile owing to the tight Ncf1-Clec4b interaction in mediating oxidative burst.

### 3.5. Clec4b Regulates Inflammatory Status of Innate Myeloid Cells Independent of Ncf1

**Figure 5:**
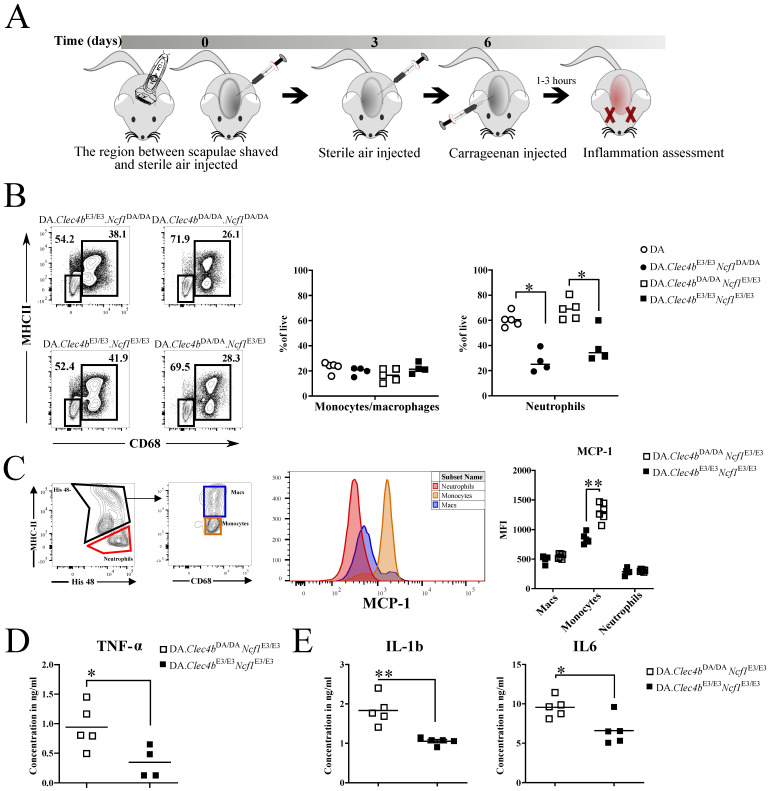
Clec4b and not Ncf1 regulate neutrophilia in the carrageenan model of acute inflammation. (**A**) Schematic of carrageenan air pouch model and injection strategy. (**B**) Flow cytometry plots depicting the gating strategy for neutrophils (Singlets/live/CD11b/c^+^/CD68^low^MHC^-^) and monocytes (Singlets/live/CD11b/c^+^/CD68^high^MHCII^+^) originating from the air pouch cells of the four rat groups, followed by a scatter plot of the monocytes and neutrophils frequencies (*n* = 4–5 rats/group). (**C**) Detection of intracellular MCP-1 in neutrophils (Singlets/live/CD11b/c+/His48+MHCII-), monocytes (Singlets/live/CD11b/c+/His48+MHCIIint) and macrophages (Singlets/live/CD11b/c+/His48+MHCII+) originating from the air pouch cells of DA.Clec4bE3/E3.Ncf1E3/E3 vs. DA.Clec4bDA/DA.Ncf1E3/E3 rats (*n* = 5–6 rats/group). Cytokines, from the air pouch exudates, measured by ELISA after 1 h (**D**) or 3 h (**E**) post carrageenan injection (*n* = 4–5 rats/group). Statistics were determined using a two-tailed Mann–Whitney U test. * *p* < 0.05; ** *p* < 0.01.

Even with the phenotype data presented above, in which Ncf1 and Clec4b interacted additively, we could not exclude that the inflammatory-promoting effects of neutrophils could be influenced by interaction between Ncf1 and Clec4b. Hence, we used an established acute model of inflammation carrageenan model of inflammation [26], to test whether Clec4b regulates neutrophil infiltration independent of Ncf1 and adaptive immunity (Figure 5A). Given the fact that most of the pouch-infiltrating cells are of myeloid origin [26], we hypothesized that Clec4b expressing cells would react differently to inflammation stimuli. Rats with Clec4bE3/E3 variant showed a 50% drop only in the frequency of neutrophils in the pouch just 3-h post carrageenan application, as compared with Clec4bDA/DA rats, irrespective of their Ncf1 genotype (Figure 5B). Compared to PBS, sorted CD11b/c cells from the carrageenan pouch had higher Clec4b expression control but no change in expression of the linked gene Mincle (Figure S2). This argues for a role of Clec4b in controlling infiltration and homing of innate cells to the site of inflammation. Monocyte chemoattractant protein-1 (MCP-1) works as a potent recruiter of monocytes/macrophages and neutrophils, and we measured the intracellular levels of MCP-1 in air pouch-infiltrating cells. Flow cytometry analysis revealed lower levels of intracellular MCP-1 only in monocytes originating from DA.Clec4bE3/E3.Ncf1E3/E3 rats, which explains the differences in neutrophil infiltration (Figure 5C). On top of that, pouch exudate from DA.Clec4bE3/E3.Ncf1E3/E3 rats, 1-h or 3-h post carrageenan, exhibited a significant decrease in pro-inflammatory cytokine concentrations (TNFα, IL1β, IL6) when compared with DA.Clec4bDA/DA.Ncf1E3/E3 rats (Figure 5D,E). Altogether, the combined data from the pouch model show that loss of Clec4b (i.e., the DA allele) correlates with increased proinflammatory cytokine production and neutrophil chemotaxis irrespective of Ncf1.

### 3.6. Clec4b Carbohydrate Recognition Motif Is Indispensable for Binding Zymosan, Leading to ROS Production

**Figure 6:**
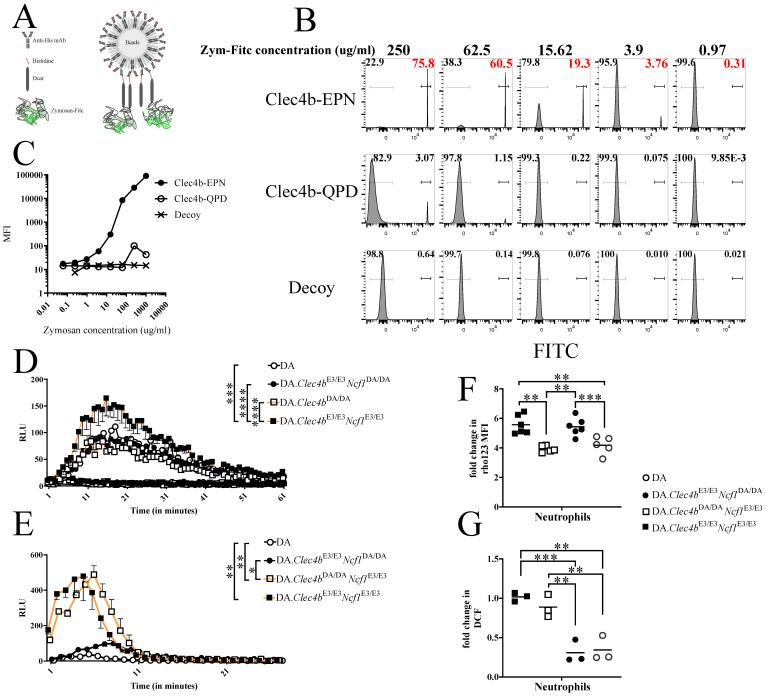
EPN motif is crucial for Clec4b-Zymosan binding resulting in ROS burst. (**A**) Schematic of Clec4b-Zymosan bead-based assay. (**B**) Flow cytometry plots depicting FITC fluorescence on beads incubated with a serial 4-fold dilution of labelled Zymosan, numbers inside the plot reflects the % of FITC positive or negative beads. (**C**) Dot plot of the MFI values coming from (**B**) at different Zymosan concentration. (**D**) Chemiluminescence detection of extracellular ROS in Zymosan-primed bone marrow cells from all rat groups (*n* = 4–6 rats/group). (**E**) Chemiluminescence detection of extracellular ROS in Fmlp-primed bone marrow cells from all rat groups (*n* = 5–6 rats/group). Neutrophils (CD11b/c^+^His48^+^) from naive bone marrow of all rat genotypes were analyzed for intracellular Zymosan-induced (**F**) (*n* = 5–6 rats/group) and Fmlp-induced (**G**) (*n* = 3 rats/group) ROS production determined by DHR assay. Data in (**D**,**E**) are presented as mean with SEM and horizontal lines in (**F**,**G**) represent mean values. Statistics in (**D**–**G**) determined using one-way analysis of variance with Tukey’s multiple comparison test. * *p* < 0.05, ** *p* < 0.01; *** *p* < 0.001, **** *p* < 0.0001.

In light of our data and backed by published reports on the ability of CLRs in recognizing carbohydrate residues [33,34], we tested whether Clec4b bind glycans. Zymosan, a glycan extract from the yeast cell wall, has been shown to be a ligand for toll-like receptor-2 and Dectin-1 and also a strong inducer of ROS [35,36]. Moreover, a tripeptide motif in CLR’s carbohydrate recognition domain dictates the specificity towards carbohydrate moieties. For instance, an EPN (Glu-Pro-Asn) motif potentially binds glucose/mannose residues whereas a QPD (Gln-Pro-Asp) binds galactose [37]. Therefore, we produced two versions of recombinant rat Clec4b protein: one unmodified (Clec4b-EPN) and the other modified version where we mutated the sugar-binding motif from EPN→QPD (Clec4b-QPD). Additionally, we produced an unrelated decoy protein to control for unspecific binding. We further assessed the ability of Clec4b to bind Zymosan in a flow cytometry bead-based assay [27] (Figure 6A). Binding kinetics plotted using the mean fluorescence intensity (MFI) of recombinant rat Clec4b protein to Zymosan, showed a specific binding compared to the decoy protein following a 4-fold serial dilution of Zymosan-FITC labeled bio-particles (Figure 6B). Strikingly, the signal was lost in the modified Clec4b-QPD, indicating that the Zymosan binding to Clec4b entails an EPN motif (Figure 6C). Next, we examined whether ROS production was affected by the Clec4b deficiency in DA animals. Indeed, cells originating from rats that harbor a functional Clec4b (DA.Clec4bE3/E3.Ncf1DA/DA and DA.Clec4bE3/E3.Ncf1E3/E3) achieved a higher ROS burst both extracellularly (1.5 times higher) and intracellularly (2-fold increase) (Figure 6D,F). Conversely, BM cells stimulated with N-Formylmethionyl-leucyl-phenylalanine (Fmlp), a specific ligand for formyl peptide receptor (FPR), resulted in a Clec4b-independent burst (Figure 6E,G). Lastly, we observed an increased Clec4b expression on BM cells from DA.Clec4bE3/E3 rats after ex vivo Zymosan stimulation (Figure S3). Overall, we showed that specific stimulation through Clec4b induces ROS production, and that ligation is dependent on the EPN motif.

### 3.7. Glycan Array Analysis of Glycans Binding CLEC4B

**Figure 7:**
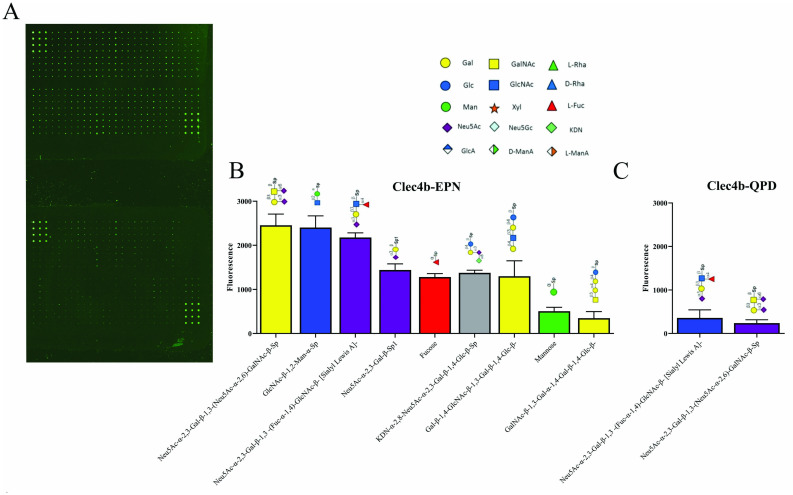
Glycan array analysis of potential Dcar ligands. (**A**) Array raw fluorescence of Clec4b-EPN (top) and Clec4b-QPD (bottom). Histidine tagged Clec4b proteins complexed with anti-Histidine biotinylated antibodies labelled with a Cy-3 coupled streptavidin was used to screen synthetic 100-glycan array. Results plotted in (**B**) and (**C**) represent hits that could only be found in Clec4b-EPN and Clec4b-QPD, respectively. Glycans were ranked according to their fluorescence. Error bars represent the SD of technical quadruplicates.

The sugar binding profile of Clec4b remains nebulous. Therefore, we generated C-terminal His-tag Clec4b-EPN and Clec4b-QPD proteins that could be detected with a fluorescently labelled anti-His antibody for the screening of 100-glycans bearing array (Figure 7A). Using an irrelevant protein, we identified Clec4b-specific interactions. 6 out of the 9 positive hits included at least one galactose residue and 4 of them harbor a N-acetylneuraminic acid (Neu5Ac). The data suggest that the position of the galactose may affect the binding as most of the positive hits contained the galactose residue in the middle or at the end of the glycan sequence. Moreover, we noticed a mild to weak binding to monosaccharides (fucose and mannose), yet the remaining identified hits consist of more than 2 glycan residues. Affinity to bi-, tri- or tetra- antennary glycans was not discussed here because the array used did not contain any (Figure 7B). Alas, we did not observe a switch in glycan affinity between Dcar-EPN and Dcar-QPD (glucose→mannose), but fewer positive hits and a relatively weaker signal in general (Figure 7C). Accordingly, these results indicate that rat Clec4b may possess certain affinity towards galactose bound sialic acids.

## 4. Discussion

Based on a recent discovery that a major locus associated with arthritis regulate the expression levels of the Clec4b protein, we now find that it could trigger the induction of ROS after activation with glycans and impedes neutrophilia, which are effects that could contribute to its profound regulatory control over autoimmune arthritis.

We used congenic rats to investigate the interaction between the two most important arthritis-regulating genes in rats, Clec4b and Ncf1. In the past, linkage analysis studies of PIA, using crosses between the arthritis-prone DA rats and the arthritis-resistant E3 rats, revealed 8 genome wide significant QTLs in total with a logarithm of odds (LOD) score higher than 2.8. PIA7 and PIA4 QTL were found to be the most significantly linked to arthritis severity and onset with a LOD score of 10.8 and 7.6 respectively [10]. Fine mapping of both QTL led to the positional cloning of Clec4b (PIA7) and Ncf1 (PIA4) as the underlying polymorphic genes, which were found to regulate arthritis severity in multiple rat models (CIA and PIA). Still, the nature of their interaction remains unknown, and their functional characterization is not fully conceptualized. Based on our data, an additive interaction exists between Clec4b and Ncf1 regarding PIA clinical score, incidence, and weight loss. Intracellular and extracellular ROS production followed by an unspecific stimulation, such as PMA, resulted in a similar additive phenotype. Moreover, we show that a Clec4b-specific glycan stimulus led to Ncf1/NOX2 mediated ROS production, while loss of Clec4b resulted in an expansion of neutrophils. This suggests that maintaining a low neutrophil number contributes to sustain protection against PIA.

Clec4b forms a putative pair with dendritic cell inhibitory receptor (Dcir, Clec4a), located within a conserved haplotype together with Clec4b, and a marker of dendritic cells [21]. Clec4a exists in 4 isoforms (clec4a1, clec4a2, clec4a3 and clec4a4) all of which are situated just upstream of the Clec4b gene. The loss of clec4a2 in mice has been reported to cause an expansion of DCs that leads to arthritis [21] and deficiency in clec4a4 to aggravate EAE by promoting antigen specific T cell responses [38]. Previously, we investigated whether natural polymorphisms in rat Clec4a isoforms regulate PIA, but it turned out that Clec4a-containing congenic fragments failed to influence arthritis [23]. This discrepancy could be due to species differences or to the use of gene knock-out rather than congenic fragments, in other words an effect due to linked genes or to non-physiologic deletion of the gene. Data from genetic polymorphisms, rather than genetic manipulation, are more likely to be useful for studies of complex diseases and to mimic a human scenario, at least when discussing complex diseases such as RA. Indeed, polymorphisms in Clec4a were found to be associated with RA although this could be a linked haplotype effect. Furthermore, complete deletion of a gene, versus a SNP-related functional defect will influence the disease differently. Ncf1 is a prime example of this phenomenon, in which humans that lack the Ncf1 protein will develop chronic granulomatous diseases (CGD) while an allelic variant of Ncf1 (Arg90His) drives a strong association with multiple autoimmune diseases [15]. This was first shown in rats where the susceptible and resistant strain harbored Ncf1 alleles resulting in equal protein expression yet different functional activity [6]. The prime advantage of our study lies in studying the effect of natural polymorphisms instead of gene knock-out in identifying interactions between 2 genes that are highly associated with arthritis in rodents and men.

Earlier reports have addressed the role of CLRs in promoting or demoting certain cytokines and chemokines that influence the immune system in different ways [39]. In light of these data, we investigated whether Clec4b alone exerts any control over immune cells during the priming phase of PIA. We found that Clec4b deficiency, as in the DA rat, resulted in Ncf1 independent acute neutrophilia in the blood and different lymphoid organs (spleen and LN) of DA. Our findings were reproduced in an acute model of inflammation where neutrophil infiltration was dependent on functional expression of Clec4b. Furthermore, we found that the Clec4b polymorphism regulates prominent chemokines (MCP-1, MIP1a, and MIP2) that are implicated in neutrophil chemotaxis. In an earlier report it has been shown that mouse Clec4b1 (mDCAR1), enhanced chemokines and cytokine production and Th1 immunity [40]. This discrepancy could be because the mouse possesses 2 closely related clec4b genes (clec4b1 and clec4b2); while the rats possess only one clec4b gene and that evolutional divergence may have a functional effect. Since Clec4b is an ITAM-bearing receptor, it was believed to be a positive regulator on immune cells [41]. However, recent reports demonstrate that some ITIM-bearing receptors work as activators rather than inhibitors, while other ITAM-bearing receptors provide regulatory functions [20,42]. Nevertheless, our data show that activation of Clec4b inhibits, rather than activates, inflammatory response, and Clec4b abrogation dramatically drives a hyperinflammatory status in neutrophils. This neutrophils-restricted effect comes in addition to the role of Clec4b+CD4+ dendritic cells in prohibiting the activation of autoreactive bystander T cells during priming [23].

Expression of Clec4b in dendritic cells controls T cell activation [23] but it is also likely that its expression in neutrophils regulate innate immune mechanisms. Both the dendritic cell mediated, and the neutrophil mediated effect could be mediated by a Clec4b/ROS axis. To identify potential ligand that trigger Clec4b we used a bead-based strategy for flow cytometric analysis. We observed that Zymosan binds Clec4b and by changing the carbohydrate recognition domain (EPN to QPD) we could show that the activation is glycan dependent. This allowed us to examine the ROS profile in different Clec4b expressing cells using specific stimuli. To our knowledge, this is the first report that demonstrates the ability of Clec4b to mediate ROS production intracellularly and extracellularly using ligands instead of anti-Clec4b antibody [28]. Of course, Zymosan is considered as an exogenous stimulus, but it argues for a similar mechanism in vivo especially that β-glucans, which are the building blocks of Zymosan, are found abundantly on the cell wall of different fungi belonging to gut mycobiome [43]. This raises critical questions about whether Clec4b is implicated in the tolerance against our symbiotic fungi, and deserves to be addressed in the future, considering the emerging reports of microbiota-CLRs axis [44].

Induction of ROS in APC could potentially mediate the suppression of bystander autoreactive T cells, as has earlier been shown using T cell transfer models in rats [12]. It could also contribute to the presently described activation of neutrophil expansion and enhancing effect on neutrophil-mediated acute inflammation. The experiments with interaction between the Ncf1 and Clec4b alleles show that the effects are additive and promotes the arthritis along similar pathways but that do not interact epistatically. Thus, ROS from the Ncf1/NOX2 complex does not critically need Dcar to mediate its function, which is logic as there are several different pathways that could explain the Ncf1 protective effect on various inflammatory conditions [45]. Nevertheless, our data show for the first time a critical role for Dcar (Clec4b) in limiting key inflammatory mediators and in inducing ROS production, which are accountable regulatory factors for the development of arthritis.

## 5. Conclusions

In summary, the results of this study describe a novel role for *Clec4b* in mediating arthritis through the Ncf1/NOX2 axis which precipitates as an additive interaction on models of arthritis and ROS production. Furthermore, the correlation between the acute inflammation observed in the carrageenan model of inflammation and neutrophil infiltration was solely dependent on *Clec4b* expression. Meanwhile, affinity analysis of recombinantly expressed rat Dcar proteins revealed specificity to Zymosan, a component of yeast cell wall, via the carbohydrate recognition domain. This binding elicited potent Ncf1/NOX2 derived ROS production. Carbohydrate profiling analysis of wild type and mutant Dcar suggested a preferential binding to galactose bound sialic acids. Although the field of genetic interaction is dominated by plant research, we provide for the first time a report that defines the interaction between two positional cloned polymorphic SNPs associated with an autoimmune disease in eucaryotic organisms.

## Figures and Tables

**Table 1:** Protein sequence of the recombinantly expressed rat Dcar and its mutant control.

Protein Name.	Protein Sequence
The rat antigen presenting cell lectin-like receptor A1 (acc. Nb.: Q67DU9)	MPLLLLLPLLWAGALAHHHHHHGGGSENLYFQSMMEKPNRRLSELHTYNSNFTCCS DGTMVSGKVWSCCPKDWKPFGSHCYFTTDFVANWNESKEKCSHMGAHLLVIHSQE EQDFINGILDTRWGYFTGLSDQGQNQWQWIDQTPYNESVTFWHED**EPN**NDYEKCVE INHHKDIGWGWNDVVCSSEHKSICQVKKIYL
The rat antigen presenting cell lectin-like receptor A1 mutant_E167Q_N169D	MPLLLLLPLLWAGALAHHHHHHGGGSENLYFQSMMEKPNRRLSELHTYNSNFTCCS DGTMVSGKVWSCCPKDWKPFGSHCYFTTDFVANWNESKEKCSHMGAHLLVIHSQE EQDFINGILDTRWGYFTGLSDQGQNQWQWIDQTPYNESVTFWHED**QPD**NDYEKCVE INHHKDIGWGWNDVVCSSEHKSICQVKKIYL

## Data Availability

All data needed to evaluate the conclusions in the paper are present in the paper and/or the Supplementary Materials.

## Supplementary Materials

The following are available online at https://www.mdpi.com/article/10.3390/antiox11010012/s1, Figure S1: SDS-PAGE analysis of recombinant Dcar proteins, Figure S2: *Clec4b* and *Clec4e* expression analysis in cell exudates from PBS or carrageenan-induced pouch model, Figure S3: *Clec4b* kinetic expression analysis of BM cells *ex vivo* after Zymosan stimulation.

## References

[1] Smolen J.S., Aletaha D., McInnes I.B. (2016). Rheumatoid arthritis. Lancet.

[2] Manolio T.A., Collins F.S., Cox N.J., Goldstein D.B., Hindorff L.A., Hunter D.J., McCarthy M.I., Ramos E.M., Cardon L.R., Chakravarti A. (2009). Finding the missing heritability of complex diseases. Nature.

[3] Yau A.C.Y., Holmdahl R. (2016). Rheumatoid arthritis: Identifying and characterising polymorphisms using rat models. Dis. Model. Mech..

[4] Haag S., Tuncel J., Thordardottir S., Mason D.E., Yau A.C.Y., Dobritzsch D., Bäcklund J., Peters E.C., Holmdahl R. (2015). Positional identification of RT1-B (HLA-DQ) as susceptibility locus for autoimmune arthritis. J. Immunol..

[5] Rintisch C., Ameri J., Olofsson P., Luthman H., Holmdahl R. (2008). Positional cloning of the Igl genes controlling rheumatoid factor production and allergic bronchitis in rats. Proc. Natl. Acad. Sci. USA.

[6] Olofsson P., Holmberg J., Tordsson J., Lu S., Akerström B., Holmdahl R. (2003). Positional identification of Ncf1 as a gene that regulates arthritis severity in rats. Nat. Genet..

[7] Yau A.C.Y., Tuncel J., Haag S., Norin U., Houtman M., Padyukov L., Holmdahl R. (2016). Conserved 33-kb haplotype in the MHC class III region regulates chronic arthritis. Proc. Natl. Acad. Sci. USA.

[8] Lorentzen J.C., Flornes L., Eklöw C., Bäckdahl L., Ribbhammar U., Guo J.P., Smolnikova M., Dissen E., Seddighzadeh M., Brookes A.J. (2007). Association of arthritis with a gene complex encoding C-type lectin-like receptors. Arthritis Rheum..

[9] Olofsson P., Holmberg J., Pettersson U., Holmdahl R. (2003). Identification and isolation of dominant susceptibility loci for pristane-induced arthritis. J. Immunol..

[10] Olofsson P., Lu S., Holmberg J., Song T., Wernhoff P., Pettersson U., Holmdahl R. (2003). A comparative genetic analysis between collagen-induced arthritis and pristane-induced arthritis. Arthritis Rheum..

[11] Savina A., Jancic C., Hugues S., Guermonprez P., Vargas P., Moura I.C., Lennon-Duménil A.-M., Seabra M.C., Raposo G., Amigorena S. (2006). NOX2 controls phagosomal pH to regulate antigen processing during crosspresentation by dendritic cells. Cell.

[12] Gelderman K.A., Hultqvist M., Pizzolla A., Zhao M., Nandakumar K.S., Mattsson R., Holmdahl R. (2007). Macrophages suppress T cell responses and arthritis development in mice by producing reactive oxygen species. J. Clin. Investig..

[13] Deffert C., Carnesecchi S., Yuan H., Rougemont A.-L., Kelkka T., Holmdahl R., Krause K.-H., Schäppi M.G. (2012). Hyperinflammation of chronic granulomatous disease is abolished by NOX2 reconstitution in macrophages and dendritic cells. J. Pathol..

[14] Olsson L.M., Johansson Å.C., Gullstrand B., Jönsen A., Saevarsdottir S., Rönnblom L., Leonard D., Wetterö J., Sjöwall C., Svenungsson E. (2017). A single nucleotide polymorphism in the NCF1 gene leading to reduced oxidative burst is associated with systemic lupus erythematosus. Ann. Rheum. Dis..

[15] Zhao J., Ma J., Deng Y., Kelly J.A., Kim K., Bang S.Y., Lee H.-S., Li Q.-Z., Wakeland Q.-Z.L.E.K., Qiu R. (2017). A missense variant in NCF1 is associated with susceptibility to multiple autoimmune diseases. Nat. Genet..

[16] Brown G.D., Willment J.A., Whitehead L. (2018). C-type lectins in immunity and homeostasis. Nat. Rev. Immunol..

[17] Yamasaki S., Matsumoto M. (2009). C-type lectin Mincle is an activating receptor for pathogenic fungus, Malassezia. Proc. Natl. Acad. Sci. USA.

[18] Saijo S., Ikeda S., Yamabe K., Kakuta S., Ishigame H., Akitsu A., Fujikado N., Kusaka T., Kubo S., Chung S.-H. (2010). Dectin-2 recognition of α-mannans and induction of Th17 cell differentiation is essential for host defense against candida albicans. Immunity.

[19] Zhu L.L., Zhao X.Q., Jiang C., You Y., Chen X.P., Jiang Y.Y., Jia X.-M., Lin X. (2013). C-type lectin receptors dectin-3 and dectin-2 form a heterodimeric pattern-recognition receptor for host defense against fungal infection. Immunity.

[20] Gour N., Lajoie S., Smole U., White M., Hu D., Goddard P., Eng C., Mak A., Oh S., Kim J. (2018). Dysregulated invertebrate tropomyosin-dectin-1 interaction confers susceptibility to allergic diseases. Sci. Immunol..

[21] Fujikado N., Saijo S., Yonezawa T., Shimamori K., Ishii A., Sugai S., Kotaki H., Sudo K., Nose M., Iwakura Y. (2008). Dcir deficiency causes development of autoimmune diseases in mice due to excess expansion of dendritic cells. Nat. Med..

[22] Takagawa T., Kitani A., Fuss I., Levine B., Brant S.R., Peter I., Tajima M., Nakamuraand S., Strober W. (2018). An increase in LRRK2 suppresses autophagy and enhances Dectin-1-induced immunity in a mouse model of colitis. Sci. Transl. Med..

[23] Bäckdahl L., Aoun M., Norin U., Holmdahl R. (2020). Identification of Clec4b as a novel regulator of bystander activation of auto-reactive T cells and autoimmune disease. PLoS Genet..

[24] Del Fresno C., Iborra S., Saz-Leal P., Martínez-López M., Sancho D. (2018). Flexible Signaling of Myeloid C-Type Lectin Receptors in Immunity and Inflammation. Front. Immunol..

[25] Tuncel J., Haag S., Hoffmann M.H., Yau A.C.Y., Hultqvist M., Olofsson P., Backlund J., Nandakumar K.S., Weidner D., Fischer A. (2016). Animal Models of Rheumatoid Arthritis (I): Pristane-Induced Arthritis in the Rat. PLoS ONE.

[26] Duarte D.B., Vasko M.R., Fehrenbacher J.C. (2016). Models of inflammation: Carrageenan air pouch. Curr. Protoc. Pharmacol..

[27] Mccoy C.E. (2016). Flow Cytometry-Based Bead-Binding Assay for Measuring Receptor Ligand Specificity. Toll-Like Receptors.

[28] Daws M.R., Nakken B., Lobato-Pascual A., Josien R., Dissen E., Fossum S. (2019). Dendritic Cell Activating Receptor 1 (DCAR1) Associates With FcεRIγ and Is Expressed by Myeloid Cell Subsets in the Rat. Front. Immunol..

[29] Nandakumar K.S., Holmdahl R. (2006). Antibody-induced arthritis: Disease mechanisms and genes involved at the effector phase of arthritis. Arthritis Res. Ther..

[30] Wright H.L., Moots R.J., Edwards S.W. (2014). The multifactorial role of neutrophils in rheumatoid arthritis. Nat. Rev. Rheumatol..

[31] Song E., Jaishankar G.B., Saleh H., Jithpratuck W., Sahni R., Krishnaswamy G. (2011). Chronic granulomatous disease: A review of the infectious and inflammatory complications. Clin. Mol. Allergy.

[32] Zhong J., Olsson L.M., Urbonaviciute V., Yang M., Bäckdahl L., Holmdahl R. (2018). Association of NOX2 subunits genetic variants with autoimmune diseases. Free Radic. Biol. Med..

[33] Palma A.S., Feizi T., Zhang Y., Stoll M.S., Lawson A.M., Díaz-Rodríguez E., Campanero-Rhodes M.A., Costa J., Gordon S., Brown G.D. (2006). Ligands for the beta-glucan receptor, Dectin-1, assigned using “designer” microarrays of oligosaccharide probes (neoglycolipids) generated from glucan polysaccharides. J. Biol. Chem..

[34] Feinberg H., Jégouzo S.A.F., Rex M.J., Drickamer K., Weis W.I., Taylor M.E. (2017). Mechanism of pathogen recognition by human dectin-2. J. Biol. Chem..

[35] Goodridge H.S., Simmons R.M., Underhill D.M. (2007). Dectin-1 stimulation by Candida albicans yeast or zymosan triggers NFAT activation in macrophages and dendritic cells. J. Immunol..

[36] Dillon S., Agrawal S., Banerjee K., Letterio J., Denning T.L., Oswald-Richter K., Kasprowicz D.J., Kellar K., Pare J., van Dyke T. (2006). Yeast zymosan, a stimulus for TLR2 and dectin-1, induces regulatory antigen-presenting cells and immunological tolerance. J. Clin. Investig..

[37] Lee R.T., Hsu T.L., Huang S.K., Hsieh S.L., Wong C.H., Lee Y.C. (2011). Survey of immune-related, mannose/fucose-binding C-type lectin receptors reveals widely divergent sugar-binding specificities. Glycobiology.

[38] Uto T., Fukaya T., Takagi H., Arimura K., Nakamura T., Kojima N., Malissen B., Sato K. (2016). Clec4A4 is a regulatory receptor for dendritic cells that impairs inflammation and T-cell immunity. Nat. Commun..

[39] Geijtenbeek T.B.H., Gringhuis S.I. (2016). C-type lectin receptors in the control of T helper cell differentiation. Nat. Rev. Immunol..

[40] Toyonaga K., Torigoe S., Motomura Y., Kamichi T., Hayashi J.M., Morita Y.S., Noguchi N., Chuma Y., Kiyohara H., Matsuo K. (2016). C-Type Lectin Receptor DCAR Recognizes Mycobacterial Phosphatidyl-Inositol Mannosides to Promote a Th1 Response during Infection. Immunity.

[41] Ivashkiv L.B. (2009). Cross-regulation of signaling by ITAM-associated receptors. Nat. Immunol..

[42] Troegeler A., Mercier I., Cougoule C., Pietretti D., Colom A., Duval C., Manh T.-P.V., Capilla F., Poincloux R., Pingris K. (2017). C-type lectin receptor DCIR modulates immunity to tuberculosis by sustaining type I interferon signaling in dendritic cells. Proc. Natl. Acad. Sci. USA.

[43] Nash A.K., Auchtung T.A., Wong M.C., Smith D.P., Gesell J.R., Ross M.C., Stewart C.J., Metcalf G.A., Muzny D.M., Gibbs R.A. (2017). The gut mycobiome of the Human Microbiome Project healthy cohort. Microbiome.

[44] Martínez-López M., Iborra S., Conde-Garrosa R., Mastrangelo A., Danne C., Mann E.R., Reid D.M., Gaboriau-Routhiau V., Chaparro M., Lorenzo M.P. (2019). Microbiota Sensing by Mincle-Syk Axis in Dendritic Cells Regulates Interleukin-17 and -22 Production and Promotes Intestinal Barrier Integrity. Immunity.

[45] Holmdahl R., Sareila O., Olsson L.M., Bäckdahl L., Wing K. (2016). Ncf1 polymorphism reveals oxidative regulation of autoimmune chronic inflammation. Immunol. Rev..

