# Supplementary material for: Glycan Activation of Clec4b Induces Reactive Oxygen Species Protecting against Neutrophilia and Arthritis
Source: Antioxidants (Basel). 2021 Dec 22;11(1):12. doi: 10.3390/antiox11010012 (PMC8773064; doi:10.3390/antiox11010012)

Figure S1. SDS-PAGE analysis of recombinant Dcar proteins.

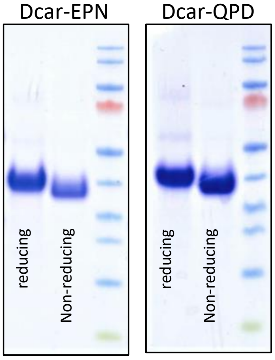

Figure S2. *Clec4b* and *Clec4e* expression analysis in cell exudates from PBS or carrageenan-induced pouch model.

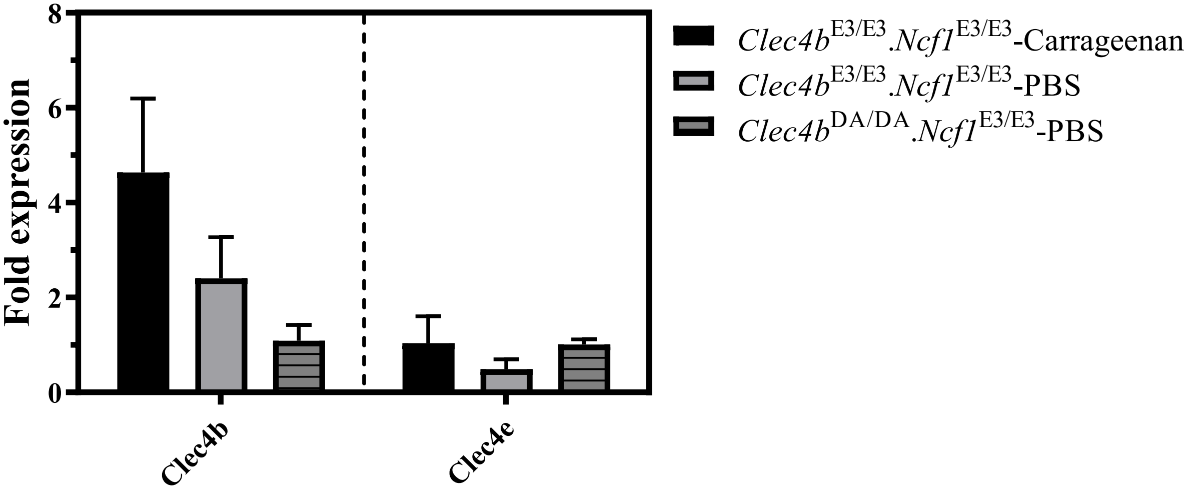

Figure S3. *Clec4b* kinetic expression analysis of BM cells ex vivo after Zymosan stimulation.

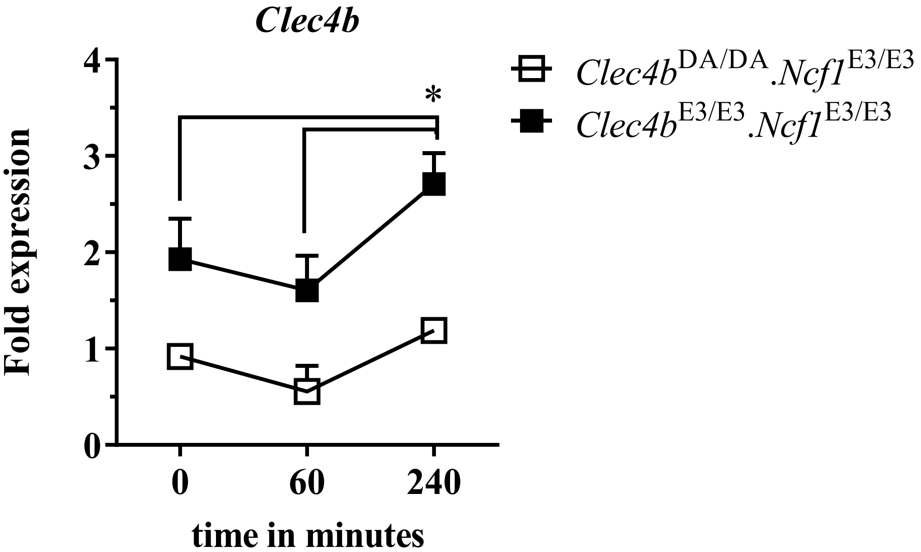

Supplement: Supplementary file 1 [file antioxidants-11-00012-s001.zip › antioxidants-1450191-supplementary.pdf]
